# A high-performance reagent-less sensor based on copper(ii) phthalocyanines supported by multi-walled carbon nanotubes for phosphate detection

**DOI:** 10.1039/d5ra00350d

**Published:** 2025-03-17

**Authors:** Malak Talbi, Adiraju Anurag, Christoph Tegenkamp, Mounir Ben Ali, Olfa Kanoun

**Affiliations:** a Professorship Measurement and Sensor Technology, Technische Universität Chemnitz Reichenhainer Str. 70 09126 Chemnitz Germany malak.talbi@etit.tu-chemnitz.de adiraju.anurag@etit.tu-chemnitz.de olfa.kanoun@etit.tu-chemnitz.de; b CRMN, Centre for Research on Microelectronics and Nanotechnology of Sousse, NANOMISENE Lab, University of Sousse LR16CRMN01 4050 Sousse Tunisia mounir.benali@issatso.rnu.tn; c University of Sousse, Higher Institute of Applied Science and Technology of Sousse 4003 Sousse Tunisia; d Analysis of Solid Surfaces, Institute for Physics, Chemnitz University of Technology 09107 Chemnitz Germany christoph.tegenkamp@physik.tu-chemnitz.de

## Abstract

Phosphate concentration is an important indicator of water quality, specifically for eutrophication levels in the presence of algae. Several analytical techniques have been proposed for phosphate monitoring, and most of them are based on indirect methods. In this study, we propose a new reagent-less direct method for the electrochemical detection of phosphate in aqueous solutions. For this, carbon screen printed electrodes (CSPE) were modified with copper(ii)-phthalocyanines (CuPc) that offer excellent oxidoreduction and electrocatalytic properties, together with chemically modified multiwalled carbon nanotubes (MWCNTs) to enhance the electrocatalytic performance of the sensor. We implemented two detection methods, which are electrochemical impedance spectroscopy (EIS) and square wave voltammetry (SWV) to compare them. The developed sensor exhibits a remarkable detection limit of 1.15 μM in the range from 10 μM to 100 μM with voltammetry and 0.13 nM in the range from 0.001 μM to 100 μM with impedance, enabling accurate measurement of phosphate concentrations in water samples. Thus, EIS shows a better sensitivity towards phosphate reduction. Furthermore, the developed sensor shows good performance in the presence of possibly interfering species that usually coexist with phosphate ions, as well as the applicability of the sensor in real water samples (tap water and nutrient water from an aquaponic system) at a good recovery rate. The electrode's response is highly reproducible with a relative standard deviation lower than 10%.

## Abstract 2

A new reagent-less method for the detection of phosphate. Carbon screen printed electrodes were modified with copper phthalocyanines offered excellent electrocatalytic properties, with carbon nanotubes to enhance the electrocatalytic performance.

## Graphical abstract

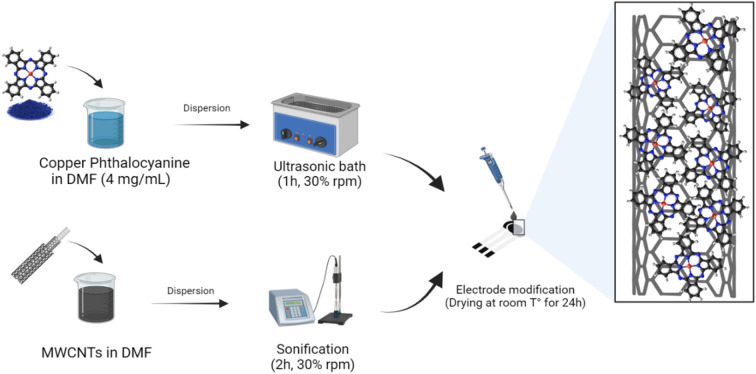

## 1. Introduction

Phosphate (P) is a widespread chemical substance, in both inorganic and organic forms, that exists in the environment (soil and water) and the human body. This nutrient is necessary for plant growth and it is widely used in agriculture fertilizers, providing vital nutrients for plants.^1,2^ Despite the significance of phosphates, high levels of this chemical can negatively affect the ecosystem; specifically, phosphate concentrations in a water body between 0.1 and 0.32 μM are defined as a threshold for a high risk of triggering harmful algal blooms and eutrophication, which can lead to the deterioration of the aquatic environment and ecosystem.^3,4^ Moreover, another concern comes from controlling phosphate concentrations in drinking water that can put human health at risk, such as kidney failure^5^ and hypophosphatemia.^6^ In this regard, maintaining acceptable phosphate concentrations is required to ensure good water quality and to protect natural water sources. According to the international standards of the World Health Organisation (WHO), a threshold of 1 mg L^−1^ of phosphate in drinking water is recommended.^7^

All these factors point out the importance of monitoring phosphate ions to safeguard the environment and water quality. In this context, enhancing current approaches and developing reliable, fast, simple, and more accurate techniques is needed. Currently, many research lines are focused on the development of analytical methods able to overcome the drawbacks of classical methods including chemiluminescence, colorimetry, fluorescence, luminescence, and capacitance measurements.^8^ Here, electrochemical methods offer a promising approach given their cost-effectiveness, simplicity of miniaturization, and high sensitivity. Many research studies have been devoted to phosphate detection based on different electrochemical approaches and innovative nanomaterials. Significant advantages of electrochemical assays include detection limit, sensitivity, selectivity, and stability. From previous investigations about phosphate detection, several electrochemical analytical methods were performed, such as sensors based on indirect detection, where different metals and associated complexes are used like silver phosphates,^9^ cobalt,^10,11^ and molybdenum.^12–14^ Also, sensors based on nanomaterial modification for offering unique chemical and physical properties, including metals,^15^ metal oxides,^16^ metallic nanoparticles,^17,18^ nanorods, and nanowires,^19^ carbon nanomaterials,^20,21^ polymers,^22,23^ and sensors employing enzymatic reactions.^24,25^ In this direction, metallophthalocyanines (MPCs) are gaining much more interest. The metal atom in the central cavity has received great interest due to its excellent electronic properties and potential applications.^26^ Furthermore, the MPCs ensure high chemical and thermal stability as well as a possibility to form highly ordered layers for the enhancement of the electrode efficiency and in particular, metal complexes, such as copper phthalocyanines (CuPc).^27^ Also, carbon nanomaterial-based sensors showed enhanced electrocatalytic activity: better selectivity, sensitivity, and lower detection limits, due to their unique electrochemical properties such as large surface-to-volume ratio.^28^ Among the different forms of carbon nanomaterials, carbon nanotubes offer good electrical conductivity, a specific surface area of up to 850 m^2^ g^−1^,^29^ chemical stability, and other outstanding electrocatalytic properties.^30^

This research is driven by the need to improve existing phosphate detection methods and explore novel approaches, considering the environmental and health implications associated with varying phosphate concentrations. In this work, a reagents-free sensor to monitor phosphate in water is developed using two complementary electrochemical techniques, voltammetry and impedance spectroscopy for ultrasensitive phosphate detection. The developed sensor is based on the functionalization of CSPE with Cu(ii)Pc and MWCNTs. The analytical performances of the sensor were evaluated by rigorously establishing satisfactory sensitivity, linear range, limit of detection, reproducibility and stability. Furthermore, the effect of additional parameters such as pH and the presence of interfering ions and real water samples spiked with phosphate showed a good recovery rate.

## 2. Materials and methods

### 2.1 Materials and apparatus

Copper(ii) phthalocyanine (C_32_H_16_CuN_8_) and carbon nanotubes were dissolved in DMF (dimethylformamide) for electrode modification. Potassium dihydrogen phosphate (KH_2_PO_4_), potassium ferrocyanide (K_4_[Fe(CN)_6_]), potassium ferricyanide (K_3_[Fe(CN)_6_]), tris(hydroxymethyl)aminomethane (NH_2_C(CH_2_OH)_3_) were used, respectively, for standard phosphates solutions, ferri/ferrocyanide redox probe solution and buffer solution. For interference measurements, sodium sulfate (Na_2_SO_4_), sodium carbonate (Na_2_CO_3_), potassium iodide (KI), sodium nitrate (NaNO_3_), calcium acetate (C_4_H_6_O_4_Ca), potassium sulfate (K_2_SO_4_) and sodium silicate (Na_2_SiO_3_). All chemicals were of analytical grade or better, purchased from Sigma-Aldrich (Germany), and applied in this study as received without further purification or refinement. All stock solutions were prepared by dissolving the desired amount of the chemical in 25 mM KHP buffer and 1 mM KCl, which was prepared in deionized water (>18.2 MΩ cm) solution unless otherwise specified. Carbon screen-printed electrodes (CSPE) and the PalmSens 4 potentiostat used in all experimental measurements have been purchased from PalmSens BV, Houten, Netherlands. Measurements of pH were carried out using a SevenExcellence pH/Cond/DO meter (S479-K) from Mettler Toledo.

### 2.2 Nanomaterial and electrode preparation

**Fig. 1:**
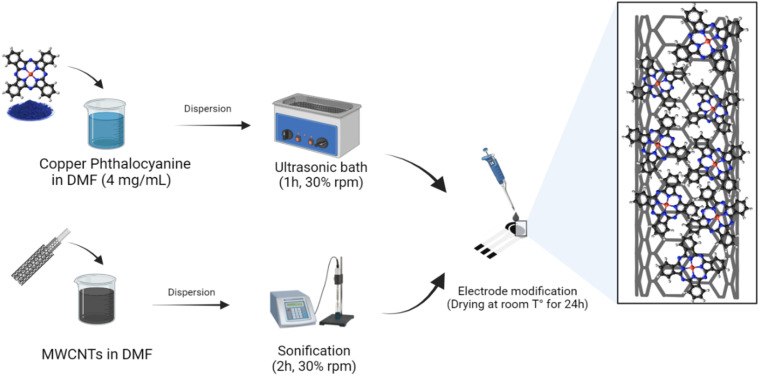
Illustration of nanomaterials and electrode preparation steps.

Copper phthalocyanines solution is prepared by dissolving 4 mg in 1 mL of DMF. Then dispersion in an ultrasonic bath for 1 hour at 30% rpm. MWCNTs have been functionalized chemically with carboxyl group –COOH groups on the surface, as reported in ^31^. First, 1.0 mg of MWCNT was treated with concentrated nitric acid and sulfuric acid solution (HNO_3_ : H_2_SO_4_) ratio 1 : 3 ratio. Second, sonication for 6 hours at 40 °C. The obtained particles were rinsed with high-purity water three times for residual acid elimination then filtered and dried. Finally, the prepared MWCNTs-COOH are dispersed in DMF solvent and ultrasonicated for 15 minutes. For the preparation of MWCNTs/CuPc/CSPE, firstly 2 μL of CuPc suspension was dropped cast on the electrode's surface and dried at ambient conditions. Then, 2 μL of MWCNTs suspension was dropped cast on top of the dried CuPC layer and left to dry for a second time at ambient conditions. The preparation steps of MWCNTs/CuPc/CSPE are described in Fig. 1.

## 3. Results and discussion

### 3.1 Structural and morphological characterization

#### 3.1.1 Optical characterization

**Fig. 2:**
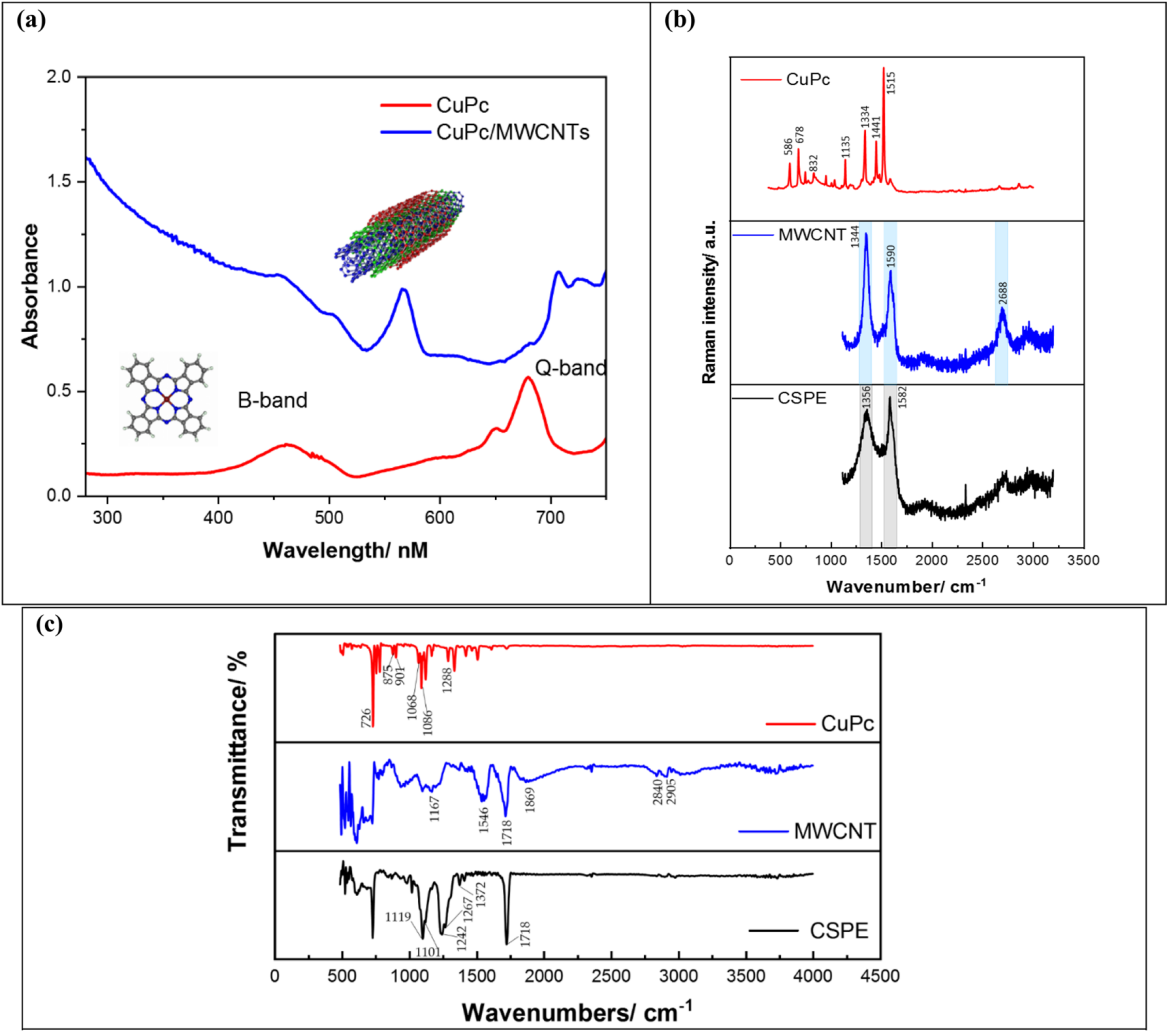
(a) Ultraviolet-visible absorption spectra of copper phthalocyanine and multi-walled carbon nanotube solution, (b) Raman spectra, (c) FTIR spectra of CuPc, MWCNT and bare CSPE.

Optical absorption spectroscopy is used to characterize predominant phases of the copper phthalocyanine and it is an appropriate method to identify the interaction between the copper phthalocyanine and carbon nanotube. The optical absorption of both materials is shown in Fig. 2a. The solid-state of CuPc displayed a doublet of peaks in the visible region from 650 to 700 nm referred to as the Q-band (HOMO–LUMO transition), which represents the correspondence spectrum obtained from the dimeric and monomeric species due to π–π* transition on macrocycle of CuPc. In addition, in the near-ultraviolet region, copper phthalocyanine provides a single peak, referred to as B-band that represents the transition of orbital in the energy range from 450 to 500 nm. The position of the absorption peak is 460 nm. Also, a shift in UV-visible peaks was observed when combining CuPc and MWCNTs, which can be attributed to several factors, such as the adsorption of CuPc molecules on the MWCNT surface through the π–π* interactions, leading to charge transfer or energy transfer processes that alter the electronic states and thus the absorption characteristics. Moreover, chemical bonding between the CuPc molecules and the CNTs can appear. This bonding modifies the electronic structure of CuPc, which results in band shifts of the UV-vis spectra.

#### 3.1.2 Raman spectroscopy

Raman spectrums of bare CSPE, CuPc/CSPE, and f-MWCNT/CSPE electrodes are shown in Fig. 2b. The spectra of the bare electrode, consisting typically of amorphous carbon, show standard D (disorder) and G (graphitic) bands approximately around 1356 cm^−1^ (intensity: 800.45) and 1582 cm^−1^ (intensity: 991.15) respectively. The existence of the D band suggests the defects in the structure of graphite and the G band indicates the presence of sp^2^-bonded carbon atoms generated from the surface scattering processes.^32,33^ The Raman spectra of MWCNT consist of three main peaks. The peak at round 1344 cm^−1^ (intensity: 1283.66) is associated with the D band. While the two peaks at 1590 cm^−1^ (intensity: 988.26) and 2688 cm^−1^ (intensity: 543.40) are assigned to the G band. As explained before, the graphite bond is naturally occurring in all sp^2^ systems and second-order Raman scattering process.^34^ After the electrode's modification with CuPc, a considerable change in the Raman spectra was also observed, indicating a high density of molecules existing on the surface. Multiple peaks were identified, including bands corresponding to benzene ring creation, C–N–C vibrations, C–H bonding vibrations, pyrrole, isoindole, and C–NC bridge bonds at 586, 678, 832, 1135, 1334, 1441, and 1515 cm^−1^, respectively.^35^

#### 3.1.3 FTIR Fourier-transform infrared spectroscopy (FTIR)

Fig. 2c depicts the FTIR spectra of bare CSPE, CuPc/CSPE, and f-MWCNT/CSPE electrodes. First, the bare CSPE shows an intense peak at 1718 cm^−1^ indicating that C=C bonds are present. The peak observed at 1372 cm^−1^ corresponds to the existence of sp^3^ C–H. The alkoxy group C–O presence is indicated by the peaks at 1267 and 1242 cm^−1^. It is also described with peaks at 1119 and 1101 cm^−1^ the trans configuration of C=C bonds in the sample.^36^ Then for the CuPc/CSPE FTIR spectrum, various absorption peaks are observed. The absorption bands of the phthalocyanine characteristic observed at 875, 901, and 1068 cm^−1^ within the polymer structure, recorded the intact structural presence of phthalocyanine. The intense band at approximately 726 cm^−1^ is due to the formation of N–H stretching. The crucial C–N stretch is indicated with peaks around 1086 and 1202 cm^−1^.^27^ The FTIR spectra of the f-MWCNTs depict bonds around 2905 cm^−1^ and 2840 cm^−1^ representing the asymmetric and symmetric stretching of CH bonds. Also, at 1869 cm^−1^ the stretching of C=O of the carboxylic acid (–COOH) group is shown. At peaks around 1718 cm^−1^, 1546 cm^−1^, and 1167 cm^−1^ C=C stretching, bending deformation of O–H in –COOH and bond stretching of CO in the f-MWCNTs are seen.^34^

#### 3.1.4 Scanning electron microscope (SEM)

**Fig. 3:**
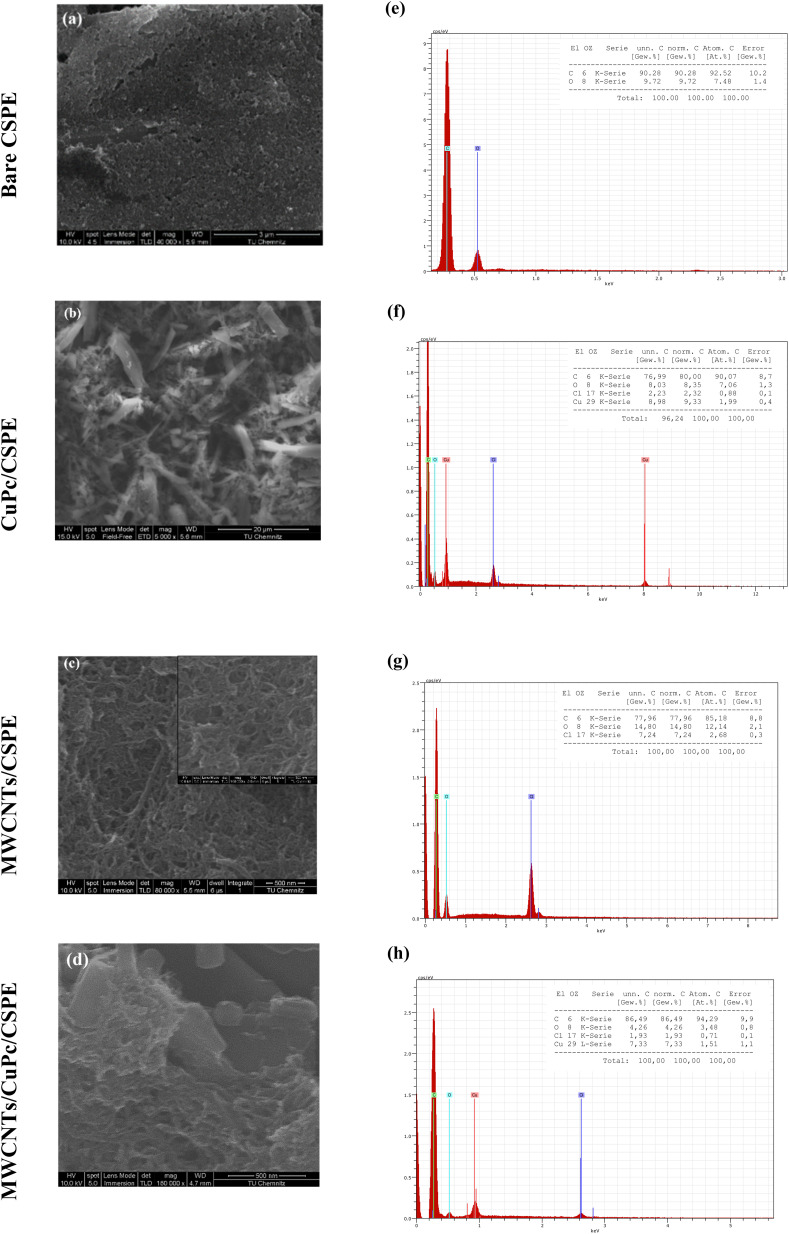
SEM images and corresponding EDX spectra for the percentages of the elemental composition of (a and e) bare CSPE; (b and f) CuPc/CSPE; (c and g) MWCNT/CSPE; (d and h) MWCNT/CuPc/CSPE.

**Fig. 4:**
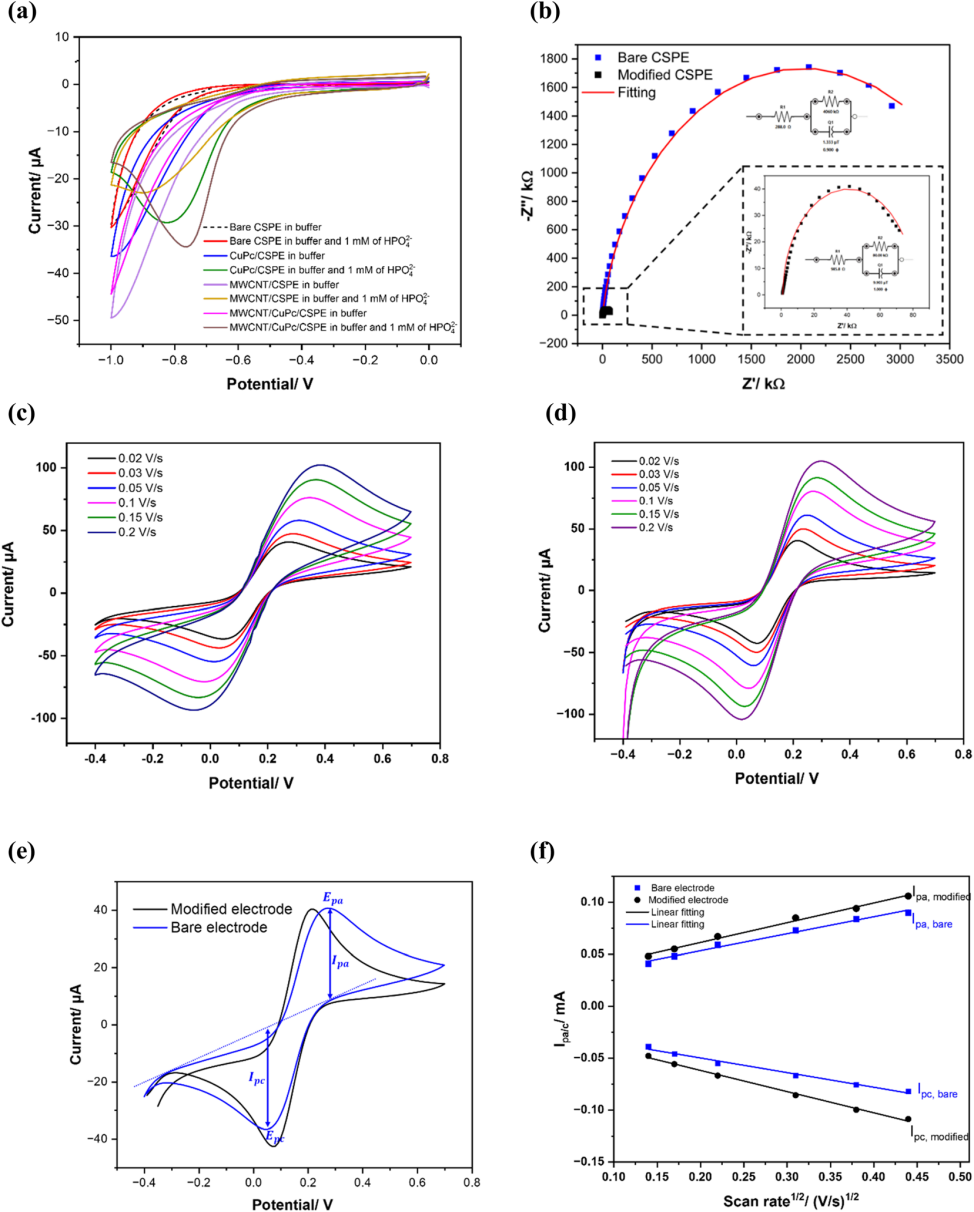
(a) Cyclic voltammograms of different material combination for electrode's modification in 1 mM of KH_2_PO_4_ and 10 mM of TRIS–HCL buffer pH 7; (b) EIS plot of bare and modified electrodes with inset of equivalent electrical circuits; (c) Bode plot; electrochemical study of ferri-ferrocyanide [Fe(CN)6]^3−/4−^ (5 mM in KCl): cyclic voltammograms at various scan rates (0.02, 0.03, 0.05, 0.1, 0.15, 0.2 V s^−1^); (d) bare CPSE; (e) MWCNTs/CuPc/CSPE; (f) oxidation-reduction peak currents as a function of the square root of the scan rate.

Fig. 4 shows the SEM images of the bare CSPE (Fig. 3a) and other different modification combinations. Fig. 3b shows that carbon nanotubes are uniformly coated on the electrode and form a spaghetti-like porous reticular formation. Moreover, the morphology and distribution of the CuPc are observed in Fig. 3c, to be dendritic. It appears that smaller, shorter nanowires have progressively aggregated to form thicker, longer, micron-sized lines.^37^ Finally, Fig. 3d displays MWCNTs and CuPc deposition of the surface of the electrode (description needed) which indicates the deposition of MWCNTs and CuPc on the surface of the electrode and provides a large surface area. EDX also was applied to study the composition of the modified electrodes and revealed the percentage of copper and carbon is higher than the bare CSPE, which directly can affect the electrochemical behavior of the modified electrode towards phosphate ions.

### 3.2 Electrochemical behavior of MWCNTs/CuPc/CSPE

Cyclic voltammetry (CV) and electrochemical impedance spectroscopy (EIS) were used to follow the stepwise modification of the CSPE surface. For the comparison, an electrode was modified only by MWCNTs (MWCNTs/CSPE) and another was modified only by CuPc (CuPc/CSPE) with a similar procedure used for modification of MWCNTs/CuPc/CSPE. To study the performance of the prepared modified electrodes, the electrochemical behavior of phosphate ions was studied using CV. Fig. 4a shows the cyclic voltammograms of CSPE with different combinations of nanomaterials in the presence and absence of phosphate ion (0.001 M of K_2_HPO_4_ at pH 7 in 25 mM KHP buffer and 1 mM KCl). The obtained voltammograms clearly show a cathodic peak corresponding to the reduction of phosphate ions, which occurred in the range of potentials from −0.9 V to −1.5 V, depending on the functionalization. The electrochemical reduction of phosphate is based on the Cu–O–P bonding between the core copper ion (Cu^2+^) in the phthalocyanine structure and goes under the following electrochemical reduction reaction:

1 HPO_4_^2−^ + 2e^−^ + H^+^ → PO_4_^3−^ + H_2_

The highest reduction current of phosphate is achieved with MWCNTs/CuPc/CSPE at the potential ≈ −1.15 V. This result is in good agreement with the EDX spectra of MWCNTs/CuPc/CSPE electrode. The large surface-to-volume ratio of the nanomaterials increases the probability of contact with phosphate ions and hence improves the electrochemical response. These results indicate that MWCNTs and CuPc significantly increase the surface area of the electrode facilitate electron transfer and enhance the current response signal of the modified electrode. The change in the interfacial electron-transfer properties on modifying the electrode surface was evaluated by EIS, as presented in Fig. 4b. The impedance Nyquist plots were recorded for bare and functionalized electrodes in 25 mM KHP buffer and 1 mM KCl at pH 7. The frequency range was swept from 0.01 Hz to 1 kHz, with 10 mV sinusoidal modulation amplitude, and by applying a polarization voltage of −0.35 V *vs.* Ag/AgCl. The absence of Warburg parts in plots indicates that the electrodes have short ion diffusion paths, providing efficient access of electrolyte ions to the surfaces of the functionalized electrodes. Randles' equivalent circuit (*R*_S_[*R*_CT_]CPE); where *R*_1_: the solution resistance; *Q*1 (CPE): the constant phase element and *R*_2_: the charge-transfer resistance; was used to fit the Nyquist plots (Fig. 4c). The fitted parameters for *R*_1_, *R*_2,_ and CPE of the equivalent circuits for the modified and bare electrodes were compared and listed in Table 1.

**Table 1:** Equivalent circuit fitted parameters for the bare and modified electrodes

Electrode	*R*_1_ (Ω)	*R*_2_ (kΩ)	CPE (μT)
Bare CSPE	268	4060	1.3_(*n*=0.900)_
MWCNTs/CuPc/CSPE	985.8	80	9.9_(*n*=1)_

The charge-transfer resistance estimated for the bare CSPE is 4060 kΩ, and steeply decreased to 80 kΩ with MWCNTs/CuPc/CSPE. It is therefore concluded that modification with MWCNTs and CuPc increases the electroactive area and consequently reduces the charge transfer resistance at the electrode/electrolyte interface. These findings correlate well with the CV measurements and suggest that the prepared material enhances the electron transfer across the supporting electrolyte and electrode. Furthermore, the ferri/ferrocyanide redox couple is used to probe the electron transfer kinetics at the chemically modified electrode surface. By analyzing the cyclic voltammetry response (peak separations, peak shapes) of the redox probe before and after surface modification with MWCNTs/CuPc, we can assess changes in the electrode kinetics induced. Fig. 4d and e illustrate the cyclic voltammograms of the bare and functionalized electrode in 5 mM ferri/ferrocyanide and 0.1 M KCl at different scan rates (0.02, 0.03, 0.05, 0.1, 0.15, 0.2 V s^−1^). The oxidation–reduction reaction of ferri/ferrocyanide redox probe occurs at both electrodes, showing clearly the modification effect. As an example, at the scan rate 0.02 V s^−1^, the peak-to-peak separation Δ*E*_p_ = (*E*_p ox_ − *E*_p red_) is calculated to be 191 mV for the bare electrode, however for the functionalized CSPE, Δ*E*_p_ was reduced to be 140 mV. This explains that the redox couple reaction is faster and more favorable with MWCNTs/CuPc/CSPE, as well as it becomes quasi-reversible, which is clearly illustrated in Fig. 4e. The quasi-reversible electron transfer process from the obtained cyclic voltammograms can be illustrated by the Randles–Sevick equation,^38^ as follows in eqn (2):2
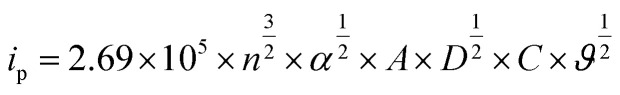
where *i*_p_ refers to the peak current, *n* is the number of electrons transferred, *A* is the electrode's area, *α* is the electron transfer coefficient, *D* is the diffusion coefficient, *ϑ* is the scan rate and *C* is the concentration of the bulk solution. After that, the anodic and cathodic peak currents were plotted as a function of the square root of the scan rate (Fig. 4f) and the correlation between them shows a linear behavior, with a correlation coefficient *R*^2^ value of 0.98 for the bare electrode and 0.99 for the modified electrode.

### 3.3 Electrocatalytic activity and phosphate measurement

#### 3.3.1 Effect of pH on phosphate sensing

**Fig. 5:**
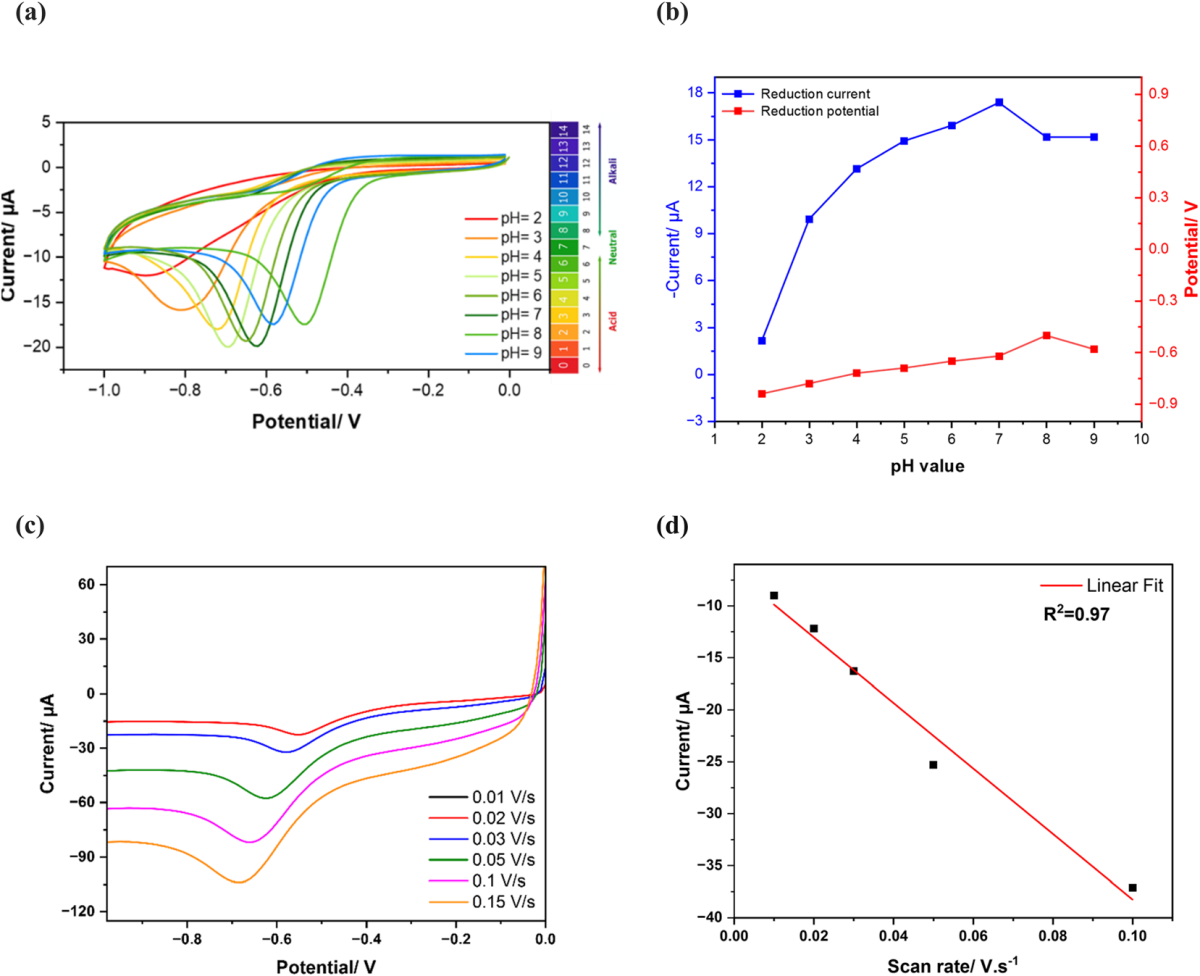
pH Study of the modified electrode with CuPc and MWCNTs in 1 mM of KH_2_PO_4_ and 25 mM of KHP KCl buffer at different pH values: (a) cyclic voltammograms of phosphate reduction at different pH values, (b) relation between phosphate reduction peak currents as a function of the pH range, (c) cyclic voltammograms of MWCNTs/CuPc/CSPE in 25 mM KHP buffer in the presence of 0.001 M of phosphate at pH 7 at different scan rates, (d) dependence between the scan rate and the cathodic phosphate reduction current.

To maximize the sensor's sensitivity towards phosphate, the investigation of the pH effect was evaluated at different pH values ranging from 2 to 9 in the presence of 1 mM of KH_2_PO_4_ in 25 mM KHP buffer and 1 mM KCl. To identify the dependence of phosphate reduction on pH, the current response is evaluated as illustrated with cyclic voltammograms in Fig. 5a. The curves confirm the existence of phosphate at different pH values with different reduction current intensities. This could be correlated to the phosphate acid–base behaviour. Aqueous phosphate could be found under several species according to the pH solution. Tri-protonated species are dominant at pH below 2.12, which is phosphoric acid (H_3_PO_4_), whereas di-protonated dominant at pH around 2.12 to 7.20, under the form of dihydrogen phosphate (H_2_PO_4_^−^), and mono-protonated species were dominant at pH around 7.20 to 12.36 under the form of hydrogen phosphate (HPO_4_^2−^). At pH higher than 12.36 the non-protonated phosphate is dominant.^39^

Based on the cyclic voltammograms in Fig. 5a, the achieved peak current was very low at pH 2, but when pH was swept from 3 to 7, the analytical response of the electrode increased gradually, hence the current increased, then decreased again when reaching pH 8 and 9. Fig. 5b shows the dependence of phosphate current reduction and potential peaks as a function of pH value, highlighting pH 7 as the optimum condition for the measurements, where the highest reduction current peak is achieved and the reduction potential slightly changes with pH.

#### 3.3.2 Effect of scan rate

To study the mass transfer mechanism at the electrode as adsorption or diffusion, cyclic voltammetry curves of MWCNTs/CuPc/CSPE were recorded with 0.001 M of phosphate in 25 mM KHP buffer at pH 7 with various scan rates ranging from 0.01 to 0.1 V s^−1^, as depicted in Fig. 5a. The cathodic peak currents exhibited linear increase with the increase of scan rate (Fig. 5b) with a correlation coefficient *R*^2^ of 0.97, following the equation (eqn (3)):

3 *i* = −315.319*ϑ* − 6.73

This finding confirms that the reaction occurring at the electrode surface is an adsorption-controlled process.^40^

### 3.4 Electrochemical detection of phosphate and analytical performances

In this section, we employed square wave voltammetry (SWV) and electrochemical impedance spectroscopy (EIS) as complementary analytical techniques to achieve a deep investigation of phosphate detection. While EIS was prioritized for its exceptional sensitivity and capacity to study interfacial charge-transfer processes at the modified electrode surface, its slower measurement speed posed limitations for dynamic monitoring. To address this, SWV was integrated as a synergistic technique, for its rapid scanning capabilities and temporal resolution to capture real-time electrochemical responses under varying analyte concentrations. This dual-method approach enabled the characterization of both the sensor's interfacial properties (*via* EIS) and its operational performance in simulated environmental conditions (*via* SWV), ensuring robust validation of detection mechanisms while maintaining practical relevance for potential field applications.

#### 3.4.1 Voltametric detection

**Fig. 6:**
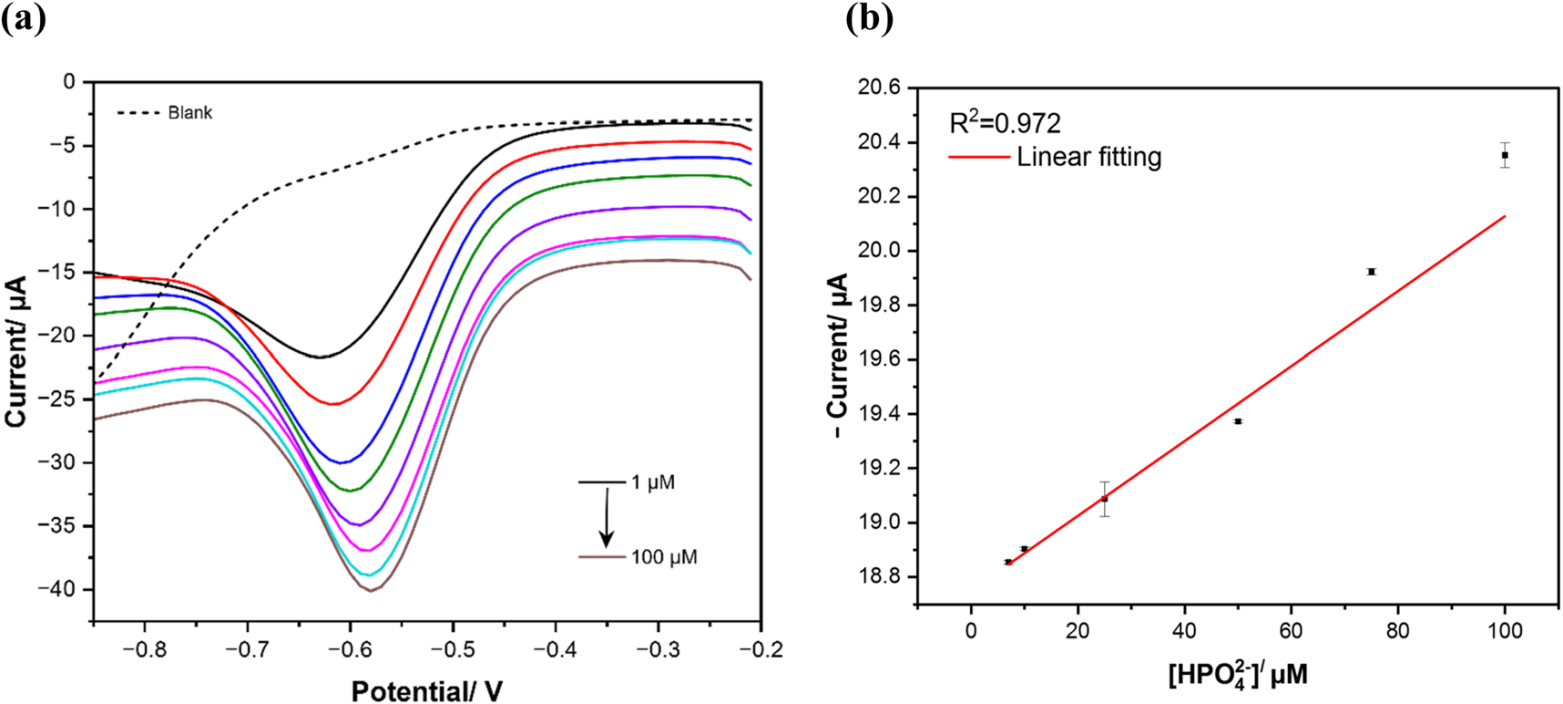
(a) Square wave voltammograms responses of MWCNTs/CuPc/CSPE in different phosphate concentrations in 25 mM KHP buffer and 1 mM KCl buffer at pH 7 and 50 mV s^−1^ scan rate; (b) calibration curve of phosphate determination in the concentration range of 10 to 100 μM (error bars indicate standard deviations over three independent measurements).

Analytical performances for phosphate detection are initially probed using square wave voltammetry (SVW) measurements, after qualitative analyses with CV. Optimum conditions for SWV were studied and applied for the phosphate detection process (*E*_pulse_ = 0.2 V, *t*_pulse_ = 0.02 s and a scan rate of 0.05 V s^−1^). SWV voltammograms illustrated in Fig. 6a show the performance of MWCNTs/CuPc/CSPE in 25 mM of KHP buffer at pH 7 under different concentrations of phosphate, ranging from 10 to 100 μM. The higher the concentration of HPO_4_^2−^, the higher the reduction peak current. This dependency of reduction phosphate current and concentrations is illustrated in the calibration curve in Fig. 6b. The analytical curve fitted with linear correlation, shows a linear dependency of phosphate concentration as a function of the reduction current, with a correlation coefficient (*R*^2^) of 0.972. The linear regression equation (eqn (4)) is then generated to be:

4 *y* (μA) = 0.013 (μM) + 18.74

After that, the sensor's figures of merits towards phosphate determination are calculated. A sensitivity of at least 0.013 μA μM^−1^ is achieved and it exhibits a low limit of detection (LOD) of 1.15 μM (for a signal-to-noise ratio 
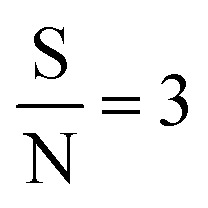
). The accomplished electrocatalytic properties of the developed sensor were evaluated in comparison to other phosphate sensors based on different modification nanomaterials and techniques reported in previous studies, as illustrated in Table 3.

#### 3.4.2 Impedimetric detection

**Fig. 7:**
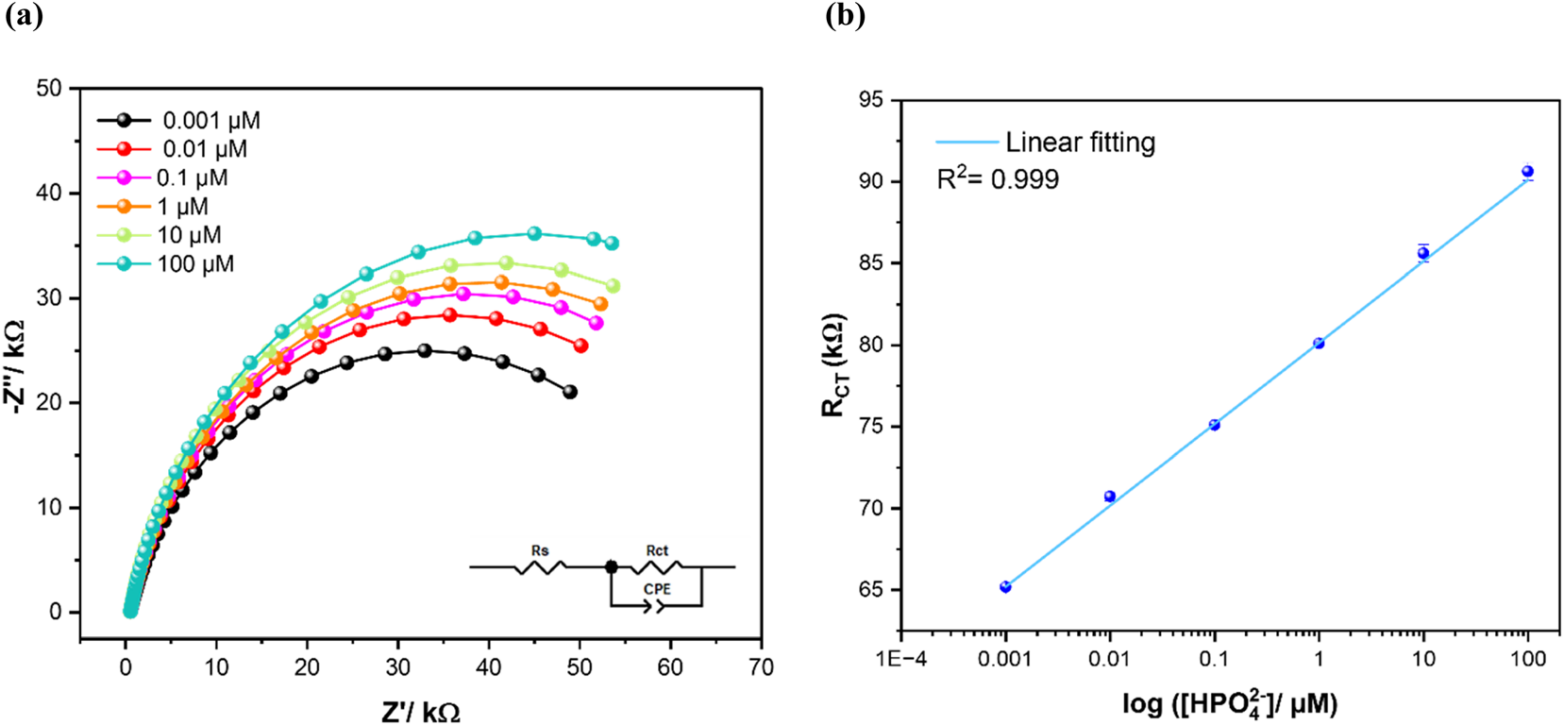
(a) Electrochemical impedance spectroscopy responses as Nyquist plots of CuPc/MWCNTs/CSPE in different concentrations of phosphate in 25 mM of KHP KCl buffer at pH 7 and 50 mV s^−1^ scan rate. The inset illustrates the electric equivalent circuit; (b) calibration curve of phosphate detection in the concentration range from 0.001 μM to 100 μM, error bars represent standard deviations of three independent measurements.

EIS was also applied to study the sensibility and selectivity of MWCNT/CuPc/CSPE towards phosphate ions. Fig. 7a represents the Nyquist diagrams of the modified sensor upon interaction with various concentrations of phosphate from 0.001 μM to 100 μM. Clearly, with the increase of HPO_4_^2−^ concentrations, the corresponding impedance value, specifically the charge transfer resistance (*R*_CT_) increases gradually (Table 2). Thus, it was selected as the parameter for the sensor response characterization. The linear variation of *R*_CT_ as a function of the logarithm of phosphate concentrations (log[HPO_4_^2−^]) is illustrated in Fig. 7b and follows the regression equation below:

5 *y* = 4.98 log([HPO_4_^2−^]) + 80.12

where *y* is *R*_CT_ in kΩ, a correlation coefficient (*R*^2^) of 0.999, and a slope of logarithmic regression value of 4.94 kΩ per decade change of concentration. The limit of detection (LOD, S/N = 3) and the limit of quantification (LOQ, S/N = 10) were calculated to be 0.31 nM and 1 nM, respectively. Therefore, the developed phosphate sensor based on MWCNT/CuPc/CSPE shows significantly good figures of merits with both detection techniques, in comparison to other phosphate sensor performances reported previously, as shown in Table 3.

**Table 2:** Equivalent circuit fitted parameters for different phosphate concentrations with the modified electrodes

Phosphate concentrations (μM)	*R*_s_ (Ω)	*R*_CT_ (kΩ)	CPE (μT)
0.001	533	65.18	55.61_(*n*=0.832)_
0.01	534.4	70.67	63.89_(*n*=0.862)_
0.1	550.5	75.00	74.31_(*n*=0.868)_
1	539.0	80.00	80.60_(*n*=0.885)_
10	495.0	85.8	84.45_(*n*=0.852)_
100	490.0	90.00	90.55_(*n*=0.850)_

**Table 3:** Comparison of the analytical performances of the proposed phosphate electrochemical sensor with previously reported for phosphate determination

Sensor	Technique	Sensitivity	Linear range	LOD (μM)	Ref.
AgNWs^a^/SPE	CV	0.71 μA μM^−1^	5 μM–1 mM	3	19
Paper CB^b^-SPE	Amperometry	0.115 μA μM^−1^	10–300 μM	4	41
Graphite SPE	CV	5.92 μA μM^−1^	0.003–0.115 μM	1.2	42
CB^b^-SPE	Amperometry	—	0.5–100 μM	0.23	43
Co_3_O_4_^c^-CC^d^	Amperometry	—	0.1–1.0 mM	10	44
			0.1–30 mM		
CuPc^e^/Au	EIS	—	0.001–1000 μM	0.008	27
PyOx^f^/Au^g^ nanowires	Amperometry	140.3 μA mM^−1^ cm^−2^	12.5–1000 μM	0.1	45
Platinum/Au Alloy nanowires arrays	Amperometry	50 mA M^−1^	248–1456 μM	45	46
Molybdenum blue/laser scribed rGO^h^	CV	4.17 μA μM^−1^ cm^−2^	1–20 μM	0.4	47
**MWCNTs/CuPc/CSPE**	**SWV**	**0.013 μA μM^−^** ^**1**^	**10–150 μM**	**1.15**	**This work**
	**EIS**	**4.94 kΩ μM^−^** ^**1**^	**0.001–100 μM**	**0.00013**	

a Silver nanowires.

b Carbon black.

c Cobalt oxide.

d Carbon cloth.

e Copper phthalocyanines.

f Pyruvate oxidase.

g Gold.

h Reduced graphene oxide.

### 3.5 Effects of interferences

**Fig. 8:**
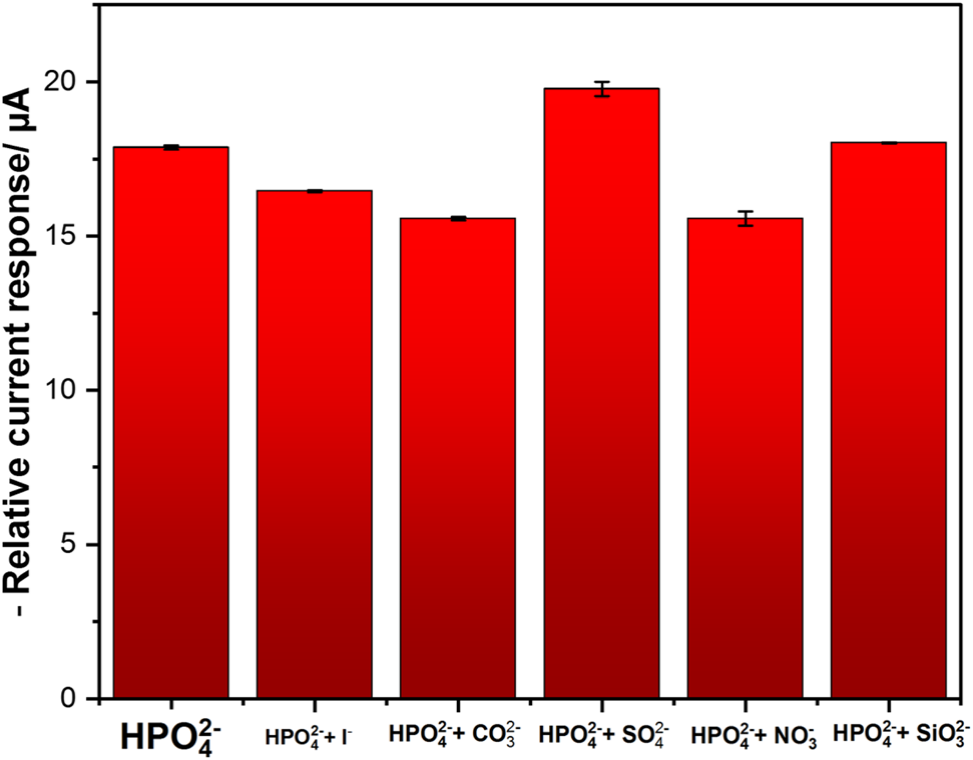
DPV interferences test; error bars represent the standard deviations of three independent measurements.

To evaluate the selectivity of the developed phosphate sensor, several ions that coexist with phosphate ions are tested. The experiments were conducted in 25 mM of KHP buffer and 1 mM KCl buffer at pH 7 containing a fixed 0.1 mM of phosphate concentration in the absence and the presence of each interfering ion at 50-fold concentration excess. Fig. 8 shows the results indicating that even at 50-fold concentrations of KI, Na_2_CO_3_, K_2_SO_4_, NaNO_3_, and Na_2_SiO_3_ did not drastically interfere with phosphate determination with a signal change of less than 10%. The interferences from NaNO_3_ is explained by the possibility of signals overlapping, because the electrochemical reduction of NO_3_^−^ occurs at a near reduction potential of phosphate. Also, for Na_2_CO_3,_ carbonate ions can compete with phosphate ions for binding on the sensor's surface. Furthermore, they can affect pH with affects the equilibrium of phosphate species. For KI slight interference, is explained by the possibility of getting adsorbed on top of the electrode's surface, hence blocking active sites for phosphate detection.^48^ These findings demonstrate that the CuPc/MWCNTs/CSPE displays good selectivity and sensitivity for phosphate determination, which strengthens the potential of the developed sensor to be used in biological and agricultural practices, where the previously identified environmental interferences are frequently observed. In addition to the interferences ions, dissolved oxygen (DO) can have a potential effect on phosphate measurements as it can undergo reduction at negative potentials following the equation (eqn (6)):

6 O_2_ + 4H^+^ + 4e^−^ → 2H_2_O

It creates a background current that overlaps with phosphate signals. To minimize this effect, electrochemical measurements should be conducted after purging the solutions with N_2_ (decrease in DO)^38^ for a certain time (±15 min). This will minimize the interference of DO to phosphate reduction signals.

### 3.6 Reproducibility and stability study

**Fig. 9:**
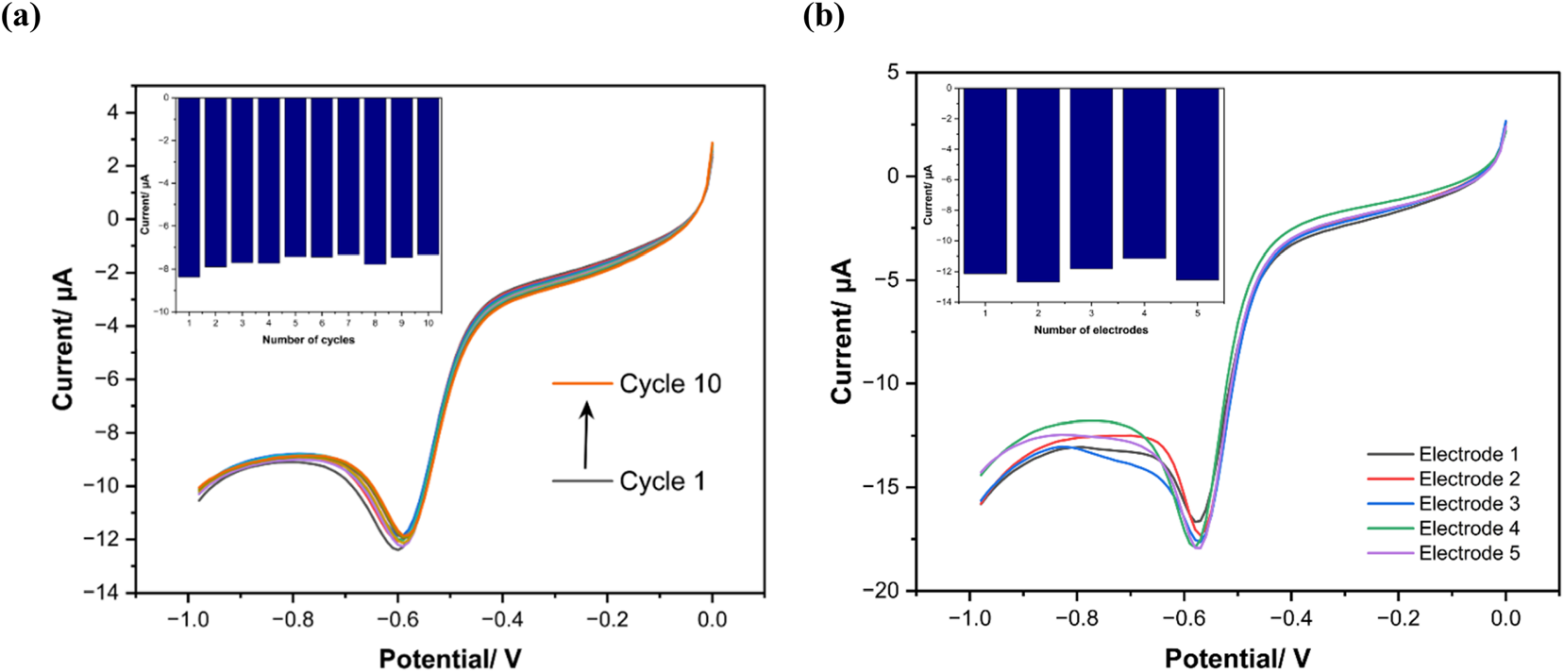
(a) CV response of 15 cycles with same modified electrode, (b) CV of 5 different sensors response prepared under same experimental conditions.

First, the reproducibility of the electrochemical response of a single modified electrode is studied by running 15 consecutive cycles with cyclic voltammetry, as depicted in Fig. 9a, in the presence of phosphate in KHP KCl buffer (25 mM, pH 7). The cyclic voltammogram shapes and respective phosphate reduction current signals remain stable after running successive measurements. Then, for the stability test of the sensors, which were prepared under the same experimental conditions (Fig. 9b), five electrodes are investigated and show an relative standard deviation (RSD) <10%. Therefore, the proposed sensor exhibits good reproducibility and stability for phosphate measurement and potentially it can make a substantial impact on many analytical applications by offering a cheaper, more commercially viable electrode for portable sensing. In future studies, testing protocols will be extended to incorporate real-time continuous monitoring systems to investigate the sensor's life which are crucial for field validation. These enhancements will complement our current multicycle stability assessments to better quantify performance degradation thresholds and failure mechanisms over extended durations.

### 3.7 Application in real sample analysis

**Fig. 10:**
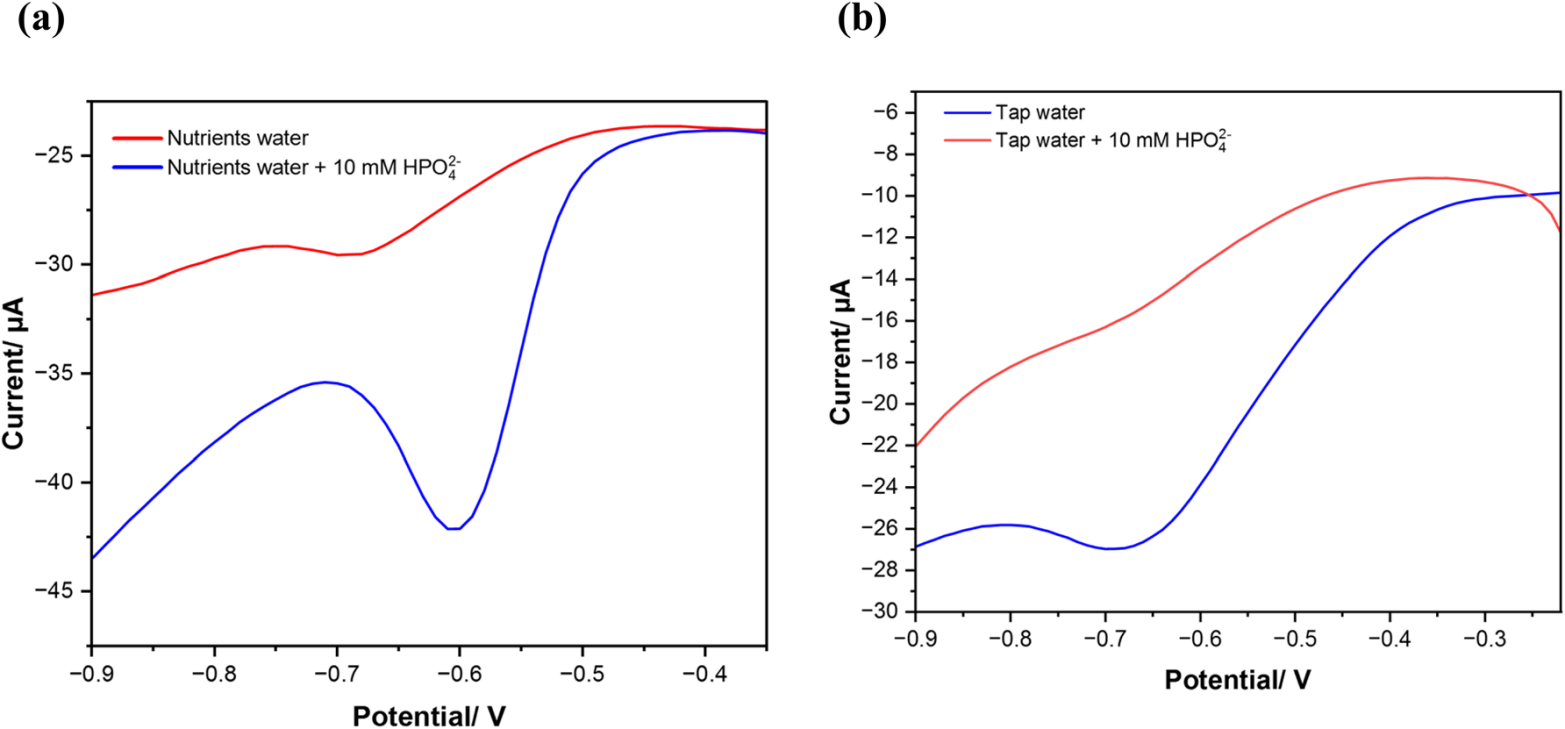
Differential wave voltammograms in the presence and absence of 10 mM of phosphate in (a) nutrient water and (b) tap water.

The developed electrode was also successfully applied for the direct determination of HPO_4_^2−^ in tap water and nutrient water samples from the aquaponic system. The major elements found in aquaponics systems are nitrogen, phosphorus, potassium, calcium, sulfur, and magnesium. Also, the presence of electroactive organic compounds poses significant challenges to the sensor's long-term performance and sensitivity. These compounds can form a fouling layer that prevents phosphate sensing and increase charge-transfer resistance. This passivation effect reduces the effective surface area available for phosphate interaction, decreasing the sensor's sensitivity over time. Moreover, some organic compounds can accelerate the degradation of the sensitive material which as well decline in its sensitivity and stability. The reliability of MWCNTs/CuPc/CSPE electrode was tested for phosphate determination by a recovery study in the different real water samples spiked with different concentrations of HPO_4_^2−^, as shown in Table 4. Before measurements, pH of the water samples was adjusted to pH 7 to meet the investigated experimental conditions. The relative recovery levels were checked by analyzing samples spiked with known quantities of phosphate (10 μM and 50 μM). Fig. 10 shows the behavior of the sensor in nutrient samples and tap water spiked with 10 mM of phosphate, where there is a significant reduction peak of phosphate. The results of this analysis show that the developed sensor can detect phosphate in real water samples with a good recovery rate.

**Table 4:** Phosphate recovery of the MWCNTs/CuPc/CSPE electrode in real water samples

Water sample	Added concentration (μM)	Found concentration (μM)	Recovery rate (%)
Tap water	10	10.6	106
	50	34.99	69.94
Nutrient's water	10	7.44	74.4
	50	57.58	115.16

## 4. Conclusion

This research demonstrated the preparation of copper(ii)-phthalocyanines (CuPc) supported by chemically functionalized multi-walled carbon nanotube (MWCNTs) deposited onto carbon screen printed electrodes (CSPE) for the determination of phosphate in water. The MWCNTs/CuPc significantly improved the electrode's electrocatalytic activity and decreased the charge transfer resistance. The developed sensor showed very good electroanalytical features toward phosphate detection with electrochemical impedance spectroscopy (EIS) and square wave voltammetry (SWV). It achieved a wide detection linear range with both techniques from 1 to 150 μM, a low limit of detection of 1.15 μM with SWV and 1 μM with EIS along with high sensitivity and selectivity in the presence of possibly interfering species that usually coexist with phosphate. In addition, the electrode's response is highly reproducible with a relative standard deviation lower than 10% observed when the same electrode is operated for consecutive measurements or when electrodes of different fabrication batches are evaluated. The applicability of the sensor in real water samples was also proven with a good recovery rate in tap water and nutrient water samples from aquaponic systems, confirming its applicability in real-world scenarios. The outcome of this study makes a notable contribution to the field of water safety analysis and has a potential impact on quality control in the water industry.

## Data availability

The data can be obtained from the corresponding author upon request.

## Conflicts of interest

There are no conflicts to declare.

## References

[cit1] Shtepliuk I. (2023). A DFT Study of Phosphate Ion Adsorption on Graphene Nanodots: Implications for Sensing. Sensors.

[cit2] Dupas R., Delmas M., Dorioz J., Garnier J. (2015). Assessing the impact of agricultural pressures on N and P loads and eutrophication risk. Ecol. Indic..

[cit3] Forano C., Farhat H., Mousty C. (2018). Recent trends in electrochemical detection of phosphate in actual waters. Curr. Opin. Electrochem..

[cit4] Wang Y., Xie X., Chen X., Huang C., Yang S. (2020). Biochar-loaded Ce 3 + -enriched ultra- fi ne ceria nanoparticles for phosphate adsorption. J. Hazard. Mater..

[cit5] Nadkarni G. N., Uribarri J. (2014). Phosphorus and the kidney: What is known and what is needed. Adv. Nutr..

[cit6] De Castro L. F., Ovejero D., Boyce A. M. (2020). Primary Disorders of Phosphate Metabolism. Eur. J. Endocrinol..

[cit7] Hitchens R. A. N. (1972). International Standards for Drinking Water, 3rd Edition. Occup. Environ. Med..

[cit8] Potdar R. P., Khollam Y. B., Shaikh S. F., Patil S. A., Al-Enizi A. M., More P. S. (2024). Europium oxide modified reduced graphene oxide composite for trace detection of hydrogen phosphate ions in soil samples. Talanta.

[cit9] Wilson A. C., Pool K. H. (1979). Potentiometric behaviour and surface composition of a prototype monohydrogenphosphate-selective electrode. Anal. Chim. Acta.

[cit10] Zeitoun R., Biswas A. (2020). Review—Potentiometric Determination of Phosphate Using Cobalt: A Review. J. Electrochem. Soc..

[cit11] Xu K., Kitazumi Y., Kano K., Shirai O. (2018). Phosphate ion sensor using a cobalt phosphate coated cobalt electrode. Electrochim. Acta.

[cit12] Arvas M. B., Gorduk O., Gencten M., Sahin Y. (2019). Preparation of a novel electrochemical sensor for phosphate detection based on a molybdenum blue modi fi ed poly (vinyl chloride) coated pencil graphite. Anal. Methods.

[cit13] Ben-Aissa S., De Marco R., Susmel S. (2023). POM@PMO plastic electrode for phosphate electrochemical detection: a further improvement of the detection limit. Microchim. Acta.

[cit14] Xu K., Wu B., Wan J., Li Y., Li M. (2022). A potentiometric phosphate ion sensor based on electrochemically modified nickel electrode. Electrochim. Acta.

[cit15] Xu K., Li Y., Li M. (2021). Potentiometric Phosphate Ion Sensor Based on Electrochemical Modified Tungsten Electrode. ACS Omega.

[cit16] Li Y., Liu J., Zhang L., Yang Q., Chen W., Wu J., Zhang L., Li X., Xu K. (2024). Amperometric Highly Sensitive Phosphate Ion Sensor Based on the Electrochemically Modified Ni Electrode. Langmuir.

[cit17] Zhang Y., Kang T., Wan Y., Chen S. (2009). Gold nanoparticles-carbon nanotubes modified sensor for electrochemical determination of organophosphate pesticides. Microchim. Acta.

[cit18] Huang X., Du D., Gong X., Cai J., Tu H., Xu X. (2008). Composite assembly of silver nanoparticles with avidin and biotinylated ache on gold for the pesticidal electrochemical sensing. Electroanalysis.

[cit19] Kabir F., Rahman T., Gurung A., Qiao Q. (2018). Electrochemical Phosphate Sensors Using Silver Nanowires Treated Screen Printed Electrodes. IEEE Sens. J..

[cit20] Sari S. R., Tsushida M., Sato T., Tominaga M. (2022). Highly sensitive detection of phosphate using well-ordered crystalline cobalt oxide nanoparticles supported by multi-walled carbon nanotubes. Mater. Adv..

[cit21] Mugo S. M., Lu W., Lemieux S. (2022). Stainless steel electrochemical capacitive microneedle sensors for multiplexed simultaneous measurement of pH, nitrates, and phosphates. Microchim. Acta.

[cit22] Nofriyani N., Manurung R. V., Debataraja A., Dwisaputra I. (2021). Phosphate ion sensor fabrication based on conductive polymer polypyrrole film coatings in doped phosphate using thick film technology. J. Mechatron. Electr. Power Veh. Technol..

[cit23] Hassani F. A. , Morley N. A., and Romero-Gonzalez M., A polymer based sensor for phosphate detection in water, 2015 IEEE SENSORS – Proceedings, 2015, pp. 1–4

[cit24] Zhang Y., Arugula M. A., Wales M., Wild J., Simonian A. L. (2014). A novel layer-by-layer assembled multi-enzyme/CNT biosensor for discriminative detection between organophosphorus and non-organophosphrus pesticides. Biosens. Bioelectron..

[cit25] Xia N., Gao Y. (2015). Carbon Nanostructures for Development of Acetylcholinesterase Electrochemical Biosensors for Determination of Pesticides. Int. J. Electrochem. Sci..

[cit26] Zagal J. H. (1992). Metallophthalocyanines reactions as catalysts in electrochemical. Coord. Chem. Rev..

[cit27] Zina F., Nooredeen N. M., Azzouzi S., Ben Ali M., Abbas M. N., Errachid A. (2017). Novel Sensitive Impedimetric Microsensor for Phosphate Detection Based on a Novel Copper Phthalocyanine Derivative Novel Sensitive Impedimetric Microsensor for Phosphate Detection Based on a Novel Copper Phthalocyanine Derivative. Anal. Lett..

[cit28] Biosensors E., Kour R., Arya S., Young S., Gupta V., Bandhoria P. (2020). Review — Recent Advances in Carbon Nanomaterials as Electrochemical Biosensors Review — Recent Advances in Carbon Nanomaterials as. J. Electrochem. Soc..

[cit29] Peigney A., Laurent C., Flahaut E., Bacsa R. R., Rousset A. (2001). Specific surface area of carbon nanotubes and bundles of carbon nanotubes. Carbon.

[cit30] Eivazzadeh-keihan R., Bahojb E., Chidar E., Jafari M. (2022). Applications of carbon-based conductive nanomaterials in biosensors. Chem. Eng. J..

[cit31] Koyun O., Gorduk S., Gencten M., Sahin Y. (2019). A novel copper(ıı) phthalocyanine-modified multiwalled carbon nanotube-based electrode for sensitive electrochemical detection of bisphenol A. New J. Chem..

[cit32] Frank O., Mohr M., Maultzsch J., Thomsen C., Riaz I., Jalil R., Novoselov K. S., Tsoukleri G., Parthenios J., Papagelis K., Kavan L., Galiotis C. (2011). Raman 2D-band splitting in graphene: Theory and experiment. ACS Nano.

[cit33] Ferrari A. C., Basko D. M. (2013). Raman spectroscopy as a versatile tool for studying the properties of graphene. Nat. Nanotechnol..

[cit34] Eswaraiah V., Sankaranarayanan V., Ramaprabhu S. (2011). Inorganic nanotubes reinforced polyvinylidene fluoride composites as low-cost electromagnetic interference shielding materials. Nanoscale Res. Lett..

[cit35] Tackley D. R., Dent G., Smith W. E. (2001). Phthalocyanines: Structure and vibrations. Phys. Chem. Chem. Phys..

[cit36] Palisoc S. T., Chua R. V. M., Natividad M. T. (2020). Highly sensitive determination of heavy metals in upland and lowland rice using AgNP/BiNP/MWCNT/nafion modified glassy carbon electrode *via* anodic stripping voltammetry. Mater. Res. Express.

[cit37] Ouyang M., Hu X., Shao X., Chen L., Li W., Bai R., Zhang L., Lv X., Tameev A., Zhang C. (2019). In situ preparation and determination of electrochemical and electrochromic properties of copper phthalocyanine-polyaniline nanocomposite films. RSC Adv..

[cit38] Prasad A., Sahu S. P., Figueiredo Stofela S. K., Chaichi A., Hasan S. M. A., Bam W., Maiti K., McPeak K. M., Liu G. L., Gartia M. R. (2021). Printed Electrode for Measuring Phosphate in Environmental Water. ACS Omega.

[cit39] Aryal R. L., Adhikari B. R., Pokhrel M. R., Poudel B. R., Paudyal H., Ghimire K. N. (2020). Preparation of pectin based adsorbent for the uptake of phosphate anion from water. Res. J. Chem. Sci..

[cit40] Elgrishi N., Rountree K. J., McCarthy B. D., Rountree E. S., Eisenhart T. T., Dempsey J. L. (2018). A Practical Beginner's Guide to Cyclic Voltammetry. J. Chem. Educ..

[cit41] Cinti S., Talarico D., Palleschi G., Moscone D., Arduini F. (2016). Novel reagentless paper-based screen-printed electrochemical sensor to detect phosphate. Anal. Chim. Acta.

[cit42] Kolliopoulos A. V., Kampouris D. K., Banks C. E. (2015). Rapid and Portable Electrochemical Quantification of Phosphorus. Anal. Chem..

[cit43] Talarico D., Arduini F., Amine A., Moscone D., Palleschi G. (2015). Screen-printed electrode modified with carbon black nanoparticles for phosphate detection by measuring the electroactive phosphomolybdate complex. Talanta.

[cit44] Xu J., Gao Z., Dou X., Song Y. Y. (2021). Needle-like Co3O4 nanoarrays as a dual-responsive amperometric sensor for enzyme-free detection of glucose and phosphate anion. J. Electroanal. Chem..

[cit45] Ogabiela E., Adeloju S. B., Cui J., Wu Y., Chen W. (2015). A novel ultrasensitive phosphate amperometric nanobiosensor based on the integration of pyruvate oxidase with highly ordered gold na- nowires array. Biosens. Bioelectron..

[cit46] Cui J., Ogabiela E. E., Hui J., Wang Y., Zhang Y., Tong L., Zhang J., Adeloju S. B., Zhang X., Wu Y. (2015). Electrochemical Biosensor based on Pt/Au Alloy Nanowire Arrays for Phosphate Detection. J. Electrochem. Soc..

[cit47] Patella B., Parisi A., Moukri N., Gitto F., Busacca A., Aiello G., Russo M., O'Riordan A., Inguanta R. (2023). Phosphate ions detection by using an electrochemical sensor based on laser-scribed graphene oxide on paper. Electrochim. Acta.

[cit48] Wang J. , Analytical Electrochemistry, 3rd edn, Wiley, 2006

